# Low-Consumption Synaptic Devices Based on Gate-All-Around InAs Nanowire Field-Effect Transistors

**DOI:** 10.1186/s11671-022-03740-1

**Published:** 2022-10-27

**Authors:** Chaofei Zha, Wei Luo, Xia Zhang, Xin Yan, Xiaomin Ren

**Affiliations:** State Key Laboratory of Information Photonics and Optical Communications, Beijing University of Posts and Telecommunications, Beijing, 100876 China

**Keywords:** InAs nanowires, Artificial synapses, Gate-all-around field-effect transistor, Synapse function

## Abstract

In this work, an artificial electronic synaptic device based on gate-all-around InAs nanowire field-effect transistor is proposed and analyzed. The deposited oxide layer (In_2_O_3_) on the InAs nanowire surface serves as a charge trapping layer for information storage. The gate voltage pulse serves as stimuli of the presynaptic membrane, and the drain current and channel conductance are treated as post-synaptic current and weights of the postsynaptic membrane, respectively. At low gate voltages, the device simulates synaptic behaviors including short-term depression and long-term depression. By increasing the amplitude and quantity of gate voltage pulses, the transition from short-term depression to long-term potentiation can be achieved. The device exhibits a large memory window of over 1 V and a minimal energy consumption of 12.5 pJ per synaptic event. This work may pave the way for the development of miniaturized low-consumption synaptic devices and related neuromorphic systems.

## Introduction

Nowadays, with the rise of artificial intelligence (AI), the computing power of current computers based on the von Neumann architecture is facing challenge. The processor and memory of computers based on von Neuman architecture are separated, which leads to low computing efficiency. Compared with von Neumann computing, neuromorphic computing based on human brain has the advantages of adaptive learning, high parallel computation, and low energy consumption [1–3]. In these days, research on neuromorphic systems that mimic the function of the human brain has made great progress in terms of materials, structures and mechanisms. Artificial synapses based on different structures, including transistors [4–6], phase-change memories [7–9] and amnestic blockers [10–12], have been developed. Compared with other structural artificial synaptic devices, field-effect transistors (FETs) have the advantages of high resistance, low noise, low power consumption, large dynamic range and easy integration, offering better variability and reliability. FETs based on III-V compound semiconductors have excellent material properties and good suppression of short-channel effects, making them one of the candidates for future high-speed applications [13–15].

One-dimensional nanowires (NWs) with high surface-volume ratio increase the possibility of electron capture, showing much stronger charge trapping effect. Most semiconductor NWs are single crystal structures with high crystal quality, which can improve device performance and extend life. Semiconductor NWs can reduce the size of the device and improve the integration degree, thus greatly reducing the power consumption of the device. Previously, dozens of silicon-based transistors were used to simulate synaptic functions [16, 17]. However, the energy consumption of synaptic devices with pure silicon materials is limited due to the circuit complexity and large-scale integration, which may cause serious power loss. InAs is a direct band gap semiconductor with a band gap of 0.36 eV. Due to the relatively narrow band gap, it has a high intrinsic carrier concentration. InAs NWs with high mobility make generated thermal electrons expected to have a long mean free path, allowing enough time to reach the traps before recombination occurs, which suitable to manufacture neural synaptic devices [18]. Currently, there has been some progress in the application of electrical stimulation on InAs NWs to simulate neural synaptic function, but the energy consumption related synaptic behavior is too large, which is not conducive to the device integration into artificial intelligence chips [19]. And mostly of them are based on top gate FETs to achieve neural synaptic functions, lacking research of gate-all-around field-effect transistors (GAA FETs). Compared with other structure, FETs with GAA structure show better performance due to their enhanced gate controllability [20].

In this paper, we proposed a novel synaptic device based on GAA FETs. The electrical properties of the device are studied by 3D Sentaurus TCAD, and the traps in oxide layer of InAs NWs are used to capture and release charges. Some basic biological synaptic properties are simulated, such as short-term depression (STD) to long-term depression (LTD). By adjusting amplitude and numbers of gate voltage pulses, the transition from STD to LTD transition is achieved. The minimum energy consumption is achieved by adjusting the state of traps in oxide layer and applied gate voltage pulses.

## Equipment Structure and Simulation Methodology

**Fig. 1:**
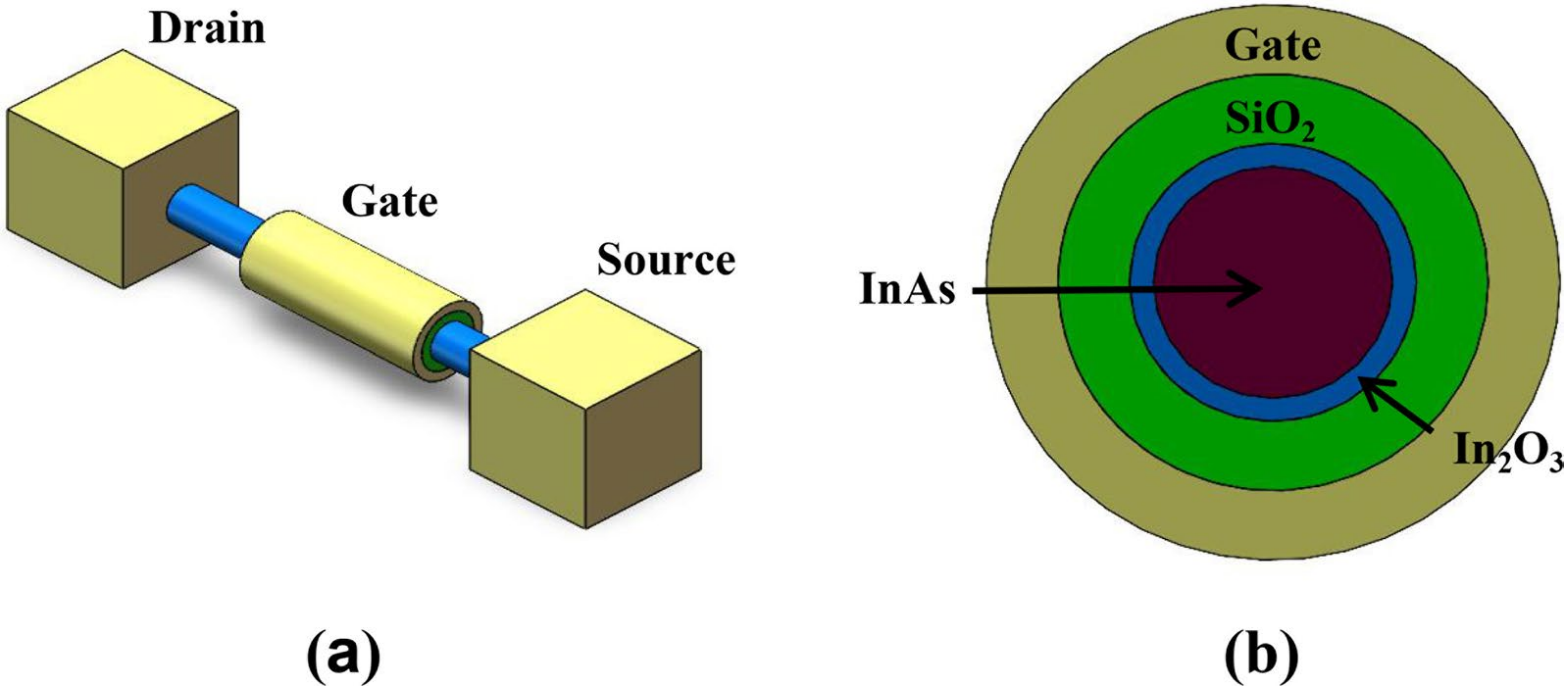
**a** Schematic diagram of a GAA InAs NW FET. **b** Cross section diagram

Figure 1a, b shows the 3D schematic diagram and 2D cross section diagram of the GAA InAs NW FET, respectively. As shown in Fig. 1b, the InAs NW is covered with an In_2_O_3_ oxide layer. In order to simulate the phenomenon that electrons may be trapped in the oxide layer, sufficient traps are introduced as trap centers to trap electrons passing through the surface barrier. The trap states come from many randomly distributed lattice traps and oxygen vacancies in the oxide layer [21, 22]. It is reported that the trap level in the oxide layer is ~ 0.5 eV above the InAs conduction band and the electron affinity of In_2_O_3_ is ~ 4.1 eV. Considering different trap density and trap energy level of In_2_O_3_ are obtained due to the introduction of neutral oxygen vacancy during fabrication process [23–26], it is necessary to discuss the range of trap energy level and concentration in our simulation. Setting exact trap energy level and concentration contribute to analyze the effect of these factors to synaptic devices. To investigate the effect of the energy level and concentration of traps on the trapping and detrapping charge of the In_2_O_3_ layer, the trap levels are uniformly set to 0.3–0.6 eV and the concentration is set to 10^17^–10^20^ cm^−3^. The GAA structure is adopted for better gate controllability, whose main parameters of the device are shown in Table 1.

**Table 1:** Main parameters of a GAA InAs NW FET

Device parameter	Value
NW diameter	20 nm
In_2_O_3_ thickness	3 nm
Gate length	0.2 μm
Gate oxide thickness	10 nm
Channel doping	10^19^ cm^−3^
Temperature	300 K
Gate work-function	5.1 eV

The fabrication process of the synaptic device could be summarized as follows. InAs NWs can be grown by molecular beam epitaxy or metal organic chemical vapor deposition. Then they are conformally coated with a 3 nm In_2_O_3_ layer and a 10 nm SiO_2_ gate dielectric by atomic layer deposition. As‐grown NWs are then transferred onto SiO_2_ substrate by using a resist-trench method [27, 28]. Metal electrodes are defined on the both ends and the middle of NW by photolithography and electromagnetic sputtering or electron beam evaporation. The contacts are exposed in EBL resist and the In_2_O_3_ and SiO_2_ coating are removed with a HF etch.

The device is simulated by 3D Sentaurus TCAD. Considering the importance of the interface to the simulation, refined grids at the interface are adopted with the size reaching 0.1 nm. The electrical properties of the device are obtained by self-consistent methods of solving the carrier continuity equation and the Poisson equation. The synaptic behavior is the result of charge generation, reorganization, trapping and detrapping in the device, thus Auger and Shockley–Read–Hall recombination models are employed to consider the production and recombination mechanisms, which is the main factor of electron motion in GAA FETs. The doping dependence model, band gap narrowing models, mobility degradation caused by impurity scattering, thermodynamic and high field models are also considered. The location of the oxide layer forming trap on the InAs NW surface is reported to store and release surface charge according to the Poole–Frenkel mechanism [29].

## Results and Discussion

**Fig. 2:**
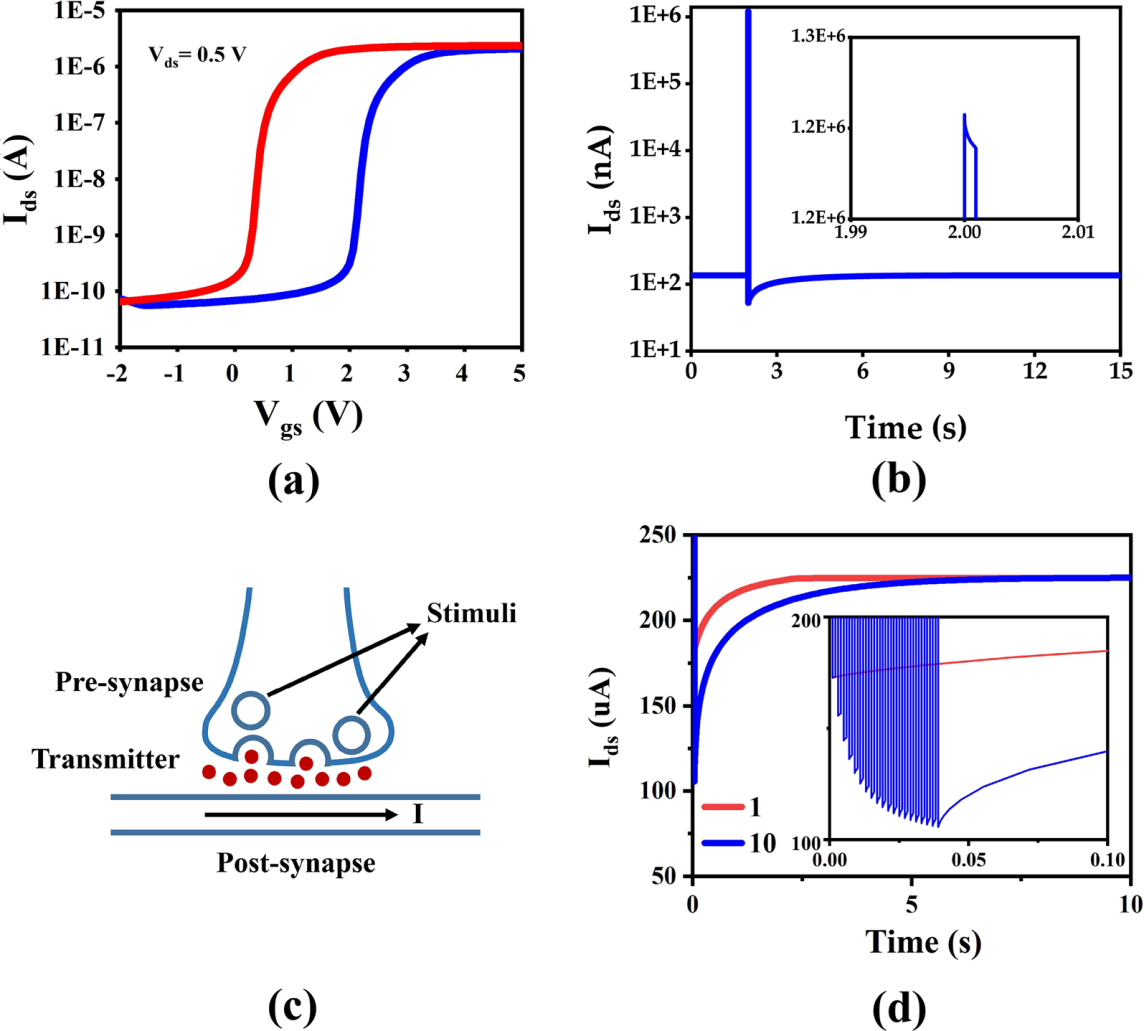
**a** Transfer characteristic of GAA InAs NW FET at *V*_ds_ = 0.5 V. **b** Change curve of drain current versus time after single pulse gate voltage of the device. The inset shows a particle enlarged view. **c** Schematic illustration of the mimicking of a biological synapse. **d** Post-synaptic current (PSC) of the device with different numbers of presynaptic spikes (1.5 V, 1 ms) at *V*_ds_ = 2 V

To simulate the influence of oxide layer traps on the electrical properties of InAs NW FET, we set the traps to be evenly placed at the energy of 0.3 eV from the conduction with the concentration of 10^19^ cm^−3^. The transmission characteristics of InAs NW FETs under the forward voltage sweep (from -2 to 5 V) and the reverse voltage sweep (from 5 to -2 V) with the drain voltage (*V*_ds_) of 0.5 V are shown in Fig. 2a. The I_on_/I_off_ ratio of the device is calculated to be about 10^4^. The mobility of InAs NWs is $2.26\times {10}^{4}\mathrm{ c}{\mathrm{m}}^{2}/\mathrm{V s}$, which increase the possibilities that hot electrons are trapped by the oxide layer, resulting in an altered threshold voltage [30]. A memory window of over 1 V is obtained due to the oxide layer of InAs NWs accommodating trapped charge to store information. Therefore, InAs NWs with deposited oxides have the potential to be used to manufactory a neuromorphic device to achieve artificial synaptic function.

The curve of drain current (*I*_ds_) versus time is shown in Fig. 2b with a gate voltage pulse of amplitude of 2 V and pulse width of 1 ms at *V*_ds_ = 0.5 V. When *V*_gs_ of 2 V is applied, the electrons in the InAs conductive channel are trapped by the oxide layer, *I*_ds_ drops continuously. When the gate voltage returns to 0 V, a part of the electrons are still captured in the trap layer, *I*_ds_ decreases significantly compared to the un-added gate voltage pulse. This is similar to the depression behavior of biological synapses, where presynaptic stimulation (2 V, 1 ms) causes changes in an excitatory post-synaptic current (PSC), and the involved charge carriers act as neurotransmitters in the synapse.

As shown in Fig. 2c, the simulation of neural networks is achieved by imitating the communication of neurons passing through synapses [31–33]. Stimulation generated by the presynaptic membrane triggers the release of neurotransmitters, which are detected by receptors in the post-synaptic membrane, causing changes in the potential and weight of the postsynaptic membrane. Similar to biological synapses, the gate voltage pulse served as stimuli of the presynaptic membrane, and the *I*_ds_ and channel conductance were treated as PSC and weights of the postsynaptic membrane, respectively. As shown in Fig. 2d, when different numbers of presynaptic spikes (1.5 V, 1 ms) are applied to the device, the memory retention time of PSC will increase significantly (from 2.8 to 7.1 s).

**Fig. 3:**
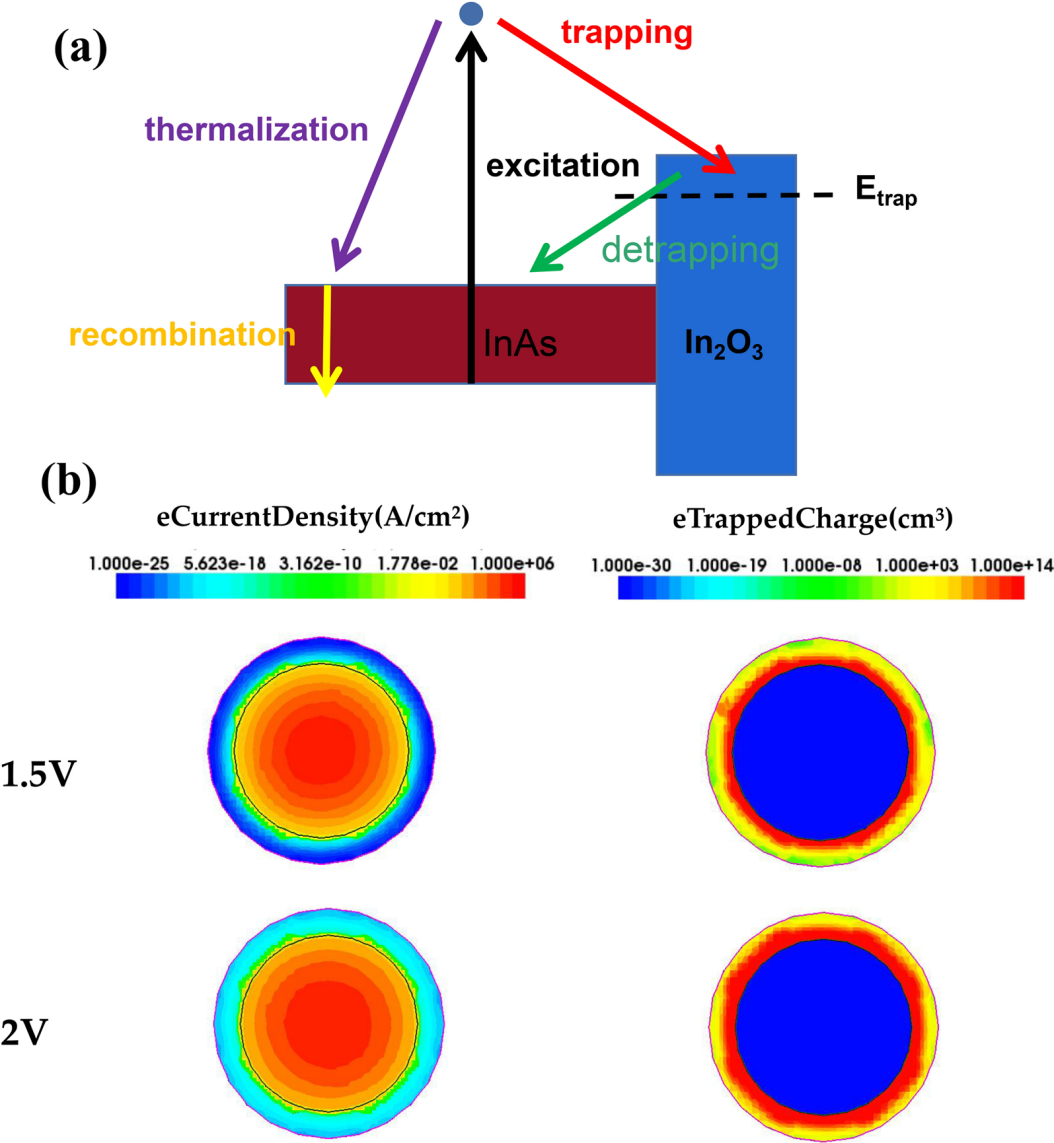
**a** Energy band diagram of excitation, recombination, thermalization, trapping and detrapping processes, and E_trap_ refer to trap level. **b** Distribution diagram of electron current density and electron trapped charge in InAs NW FETs under different gate voltages

The mechanism of charge trapping effect in NW surface can be explained by Fig. 3a, high mobility of InAs NWs offers a relatively long mean free path to thermal electrons, which leads to the increasing probability of electrons being trapped. Thus when the energy of the high energy thermal electrons generated by the gate voltage excitation in InAs NWs is above the barrier height, it is probably captured by the oxide trap layer before thermalization back to the conduction band of InAs NWs. The quantity of electrons involved in combination in the conduction band decreases. As a result, the electron density of the conduction band is reduced and the PSC decreases. Based on the retention time, synaptic plasticity can be divided into short-term plasticity and long-term plasticity, the intensity of the stimulation in our simulation can be expressed as the magnitude of the gate voltage and the quantity of gate voltage pulses. Therefore, simulating the strength of the artificial synaptic stimulus by controlling amplitude and quantity of gate voltage pulses can make the device act out synaptic behaviors. A cross-sectional diagram of the trap charge under applied different gate voltage strengths is shown in Fig. 3b. With a gate voltage of 2 V, the quantity of electrons trapped by the oxide layer is greater than that with a gate voltage of 1.5 V. The higher gate voltage reduces the barrier between the conduction band of InAs NW and the energy level of defects, making it easier for thermal electrons to overcome the barrier. As a result, the quantity of electrons in conductive channel drops, causing that PSC declines and the electrons increases in the oxide layer.

**Fig. 4:**
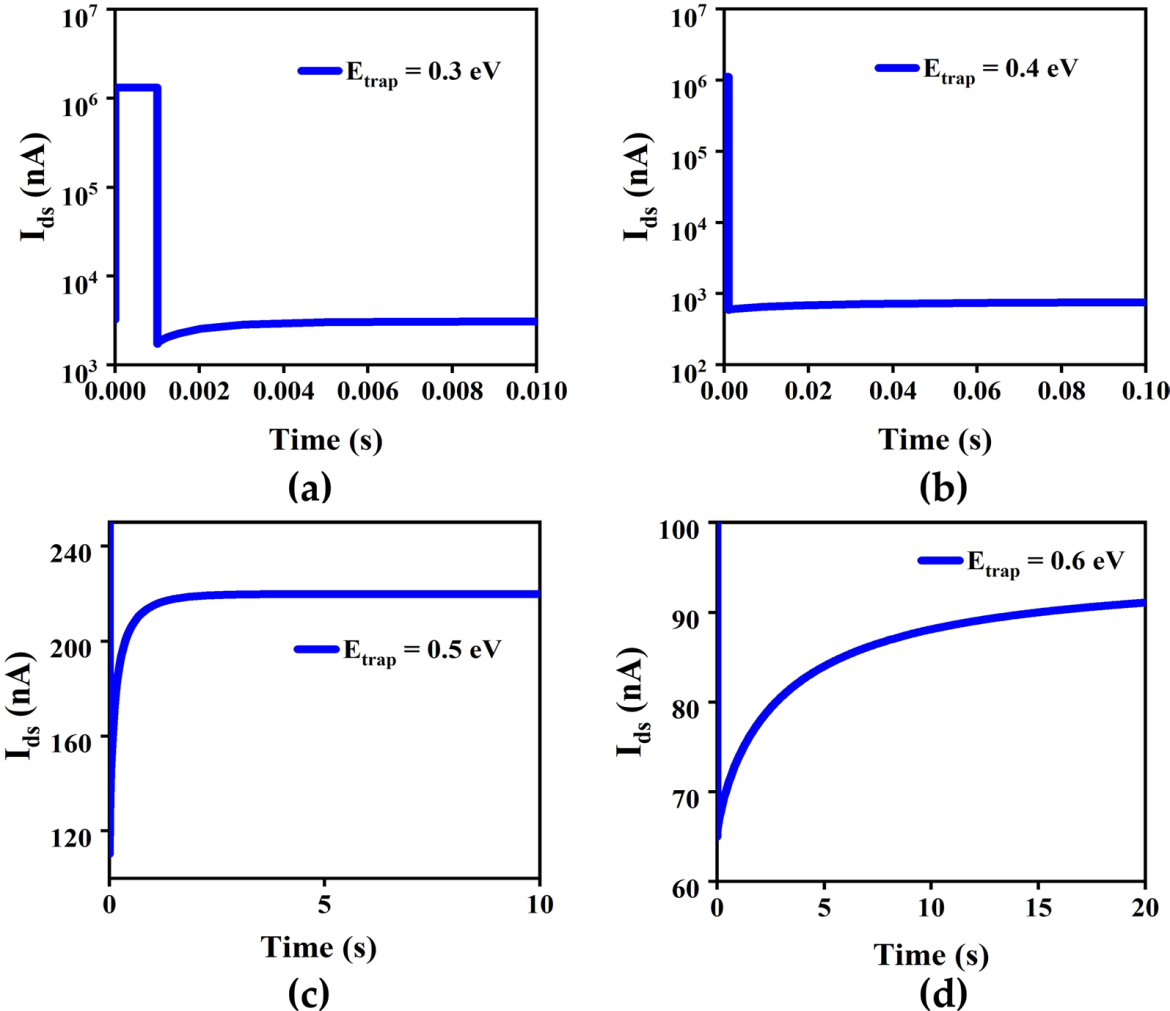
The excitatory PSC when the trap levels are set at different depth (0.3 eV, 0.4 eV, 0.5 eV, 0.6 eV) at *V*_ds_ = 0.5 V. The amplitude and width of the gate electric pulses are set at 2 V and 1 ms, respectively

In an artificial neuromorphic system, the simulation of synaptic plasticity is the cornerstone of neuromorphic devices [34, 35]. The GAA InAs NW FETs simulating synaptic plasticity mainly exploit the carrier trapping effect of oxide traps layer. The depth and concentration of traps in the oxide layer are also inconsistent due to the different growth conditions of the nanowires [23, 36]. Therefore, it is necessary to investigate the effects of different depth and concentrations of the traps in oxide layer. In the study, a gate electric pulse with amplitude of 2 V and a width of 1 ms is applied to the GAA InAs NW FET to measure current response of the device with different depth and concentrations of traps on InAs NW surface. The dependence of the trap depths and the excitatory PSC is shown in Fig. 4. The excitatory PSC at trap depths from 0.3 eV to 0.6 eV are 941 nA, 308 nA, 110 nA and 62 nA, respectively, with corresponding memory retention time of 110 ms, 0.62 s, 2.01 s and 11 s. In Sentaurus TCAD, the electron emission rate to the conduction band at the same location as the trap is

1 $${e}_{\mathrm{C}}^{n}=\frac{{v}_{\mathrm{th}}^{n}{\sigma }_{n}{\gamma }_{n}{n}_{1}}{{g}_{n}}+{e}_{\mathrm{const}}^{n}$$

where ${\gamma }_{n}$ is related to Fermi statistics, ${v}_{\mathrm{th}}^{n}$ is thermal velocity, ${\sigma }_{n}$ refers to cross sections. ${e}_{\mathrm{const}}^{n}$ represents constant emission rate and defaults to 0. ${n}_{1}={N}_{\mathrm{C}}\mathrm{exp}\left[\left({E}_{\text{trap }}-{E}_{\mathrm{C}}\right)/kT\right]$. For electrons trap, $\left({E}_{\text{trap }}-{E}_{\mathrm{C}}\right)$ is always negative, when the traps are set at a shallow level, small ${E}_{\text{trap}}$ result in a high electron emission rate, which means the trapped electrons at shallow level can detrap back into the conductive channel more easily, resulting in bigger PSC and faster memory retention time, consistent with the STD of biological synapses and rapid recovery of membrane potential. Similarly, the traps at a deep level can result in smaller excitatory PSC and slower retention time, consistent with the LTD of biological synapses and slow recovery of membrane potential.

**Fig. 5:**
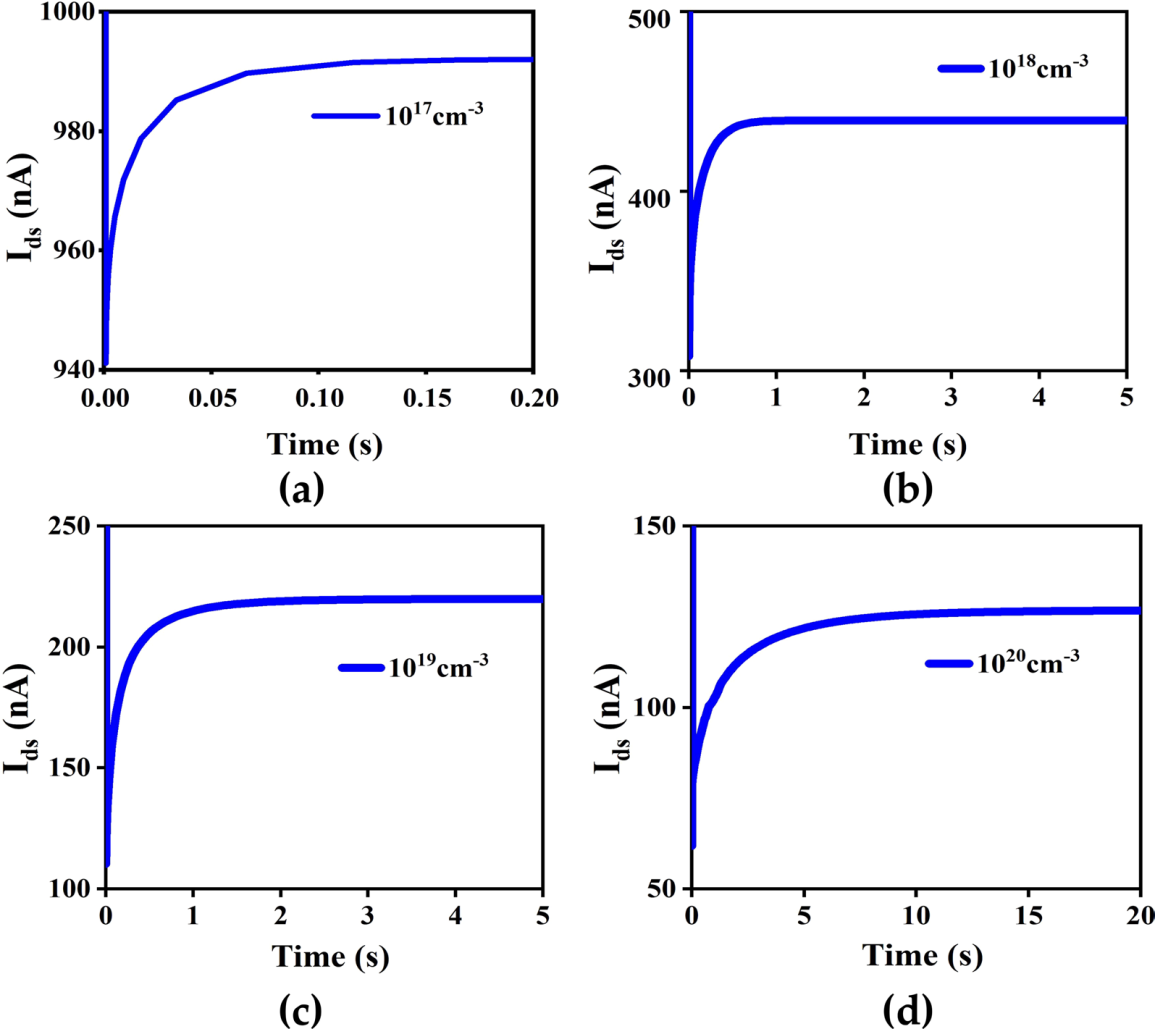
The PSC with different concentration of traps (10^17^, 10^18^, 10^19^, 10^20^ cm^−3^) in the oxide layer at *V*_ds_ = 0.5 V. The amplitude and width of the gate voltage pulses are set at 2 V and 1 ms, respectively

The dependence of the traps concentration on the excitatory PSC is shown in Fig. 5. The excitatory PSC of traps concentrations from 10^17^ to 10^20^ cm^−3^ are 1.7 μA, 0.64 μA, 110 nA and 65 nA, with corresponding memory retention time of 17.4 ms, 0.22 s, 2.01 s and 14.9 s. When the concentration of traps is low, the oxide layer traps captures relatively few electrons. As a result, *I*_ds_ increases and has a shorter retention time, corresponding to the STD of the biological synapse. Similarly, higher trap concentration result in a lower *I*_ds_ and longer retention time, corresponding to the LTD of the biological synapse. Therefore appropriate depth and concentration of traps are necessary to achieve STD and LTD. Therefore, artificially modulating the depth and concentration of oxide layer traps during growth of NWs can help devices to mimic biological synaptic behavior. Deeper energy level and higher concentrations of traps lead to difficulties in the release of trapped hot electrons in the oxide layer, causing bigger retention times for PSC when a single gate voltage pulse is applied. Increasing the quantity and the amplitude of applied gate voltage pulses resulted in insignificant changes in the retention time of the device, leading to a weakened gate controllability of the synaptic device. In order to achieve gate controllability, in our simulations, a suitable defect state with a concentration of 10^19^ cm^−3^ and a depth of 0.5 eV is selected.

**Fig. 6:**
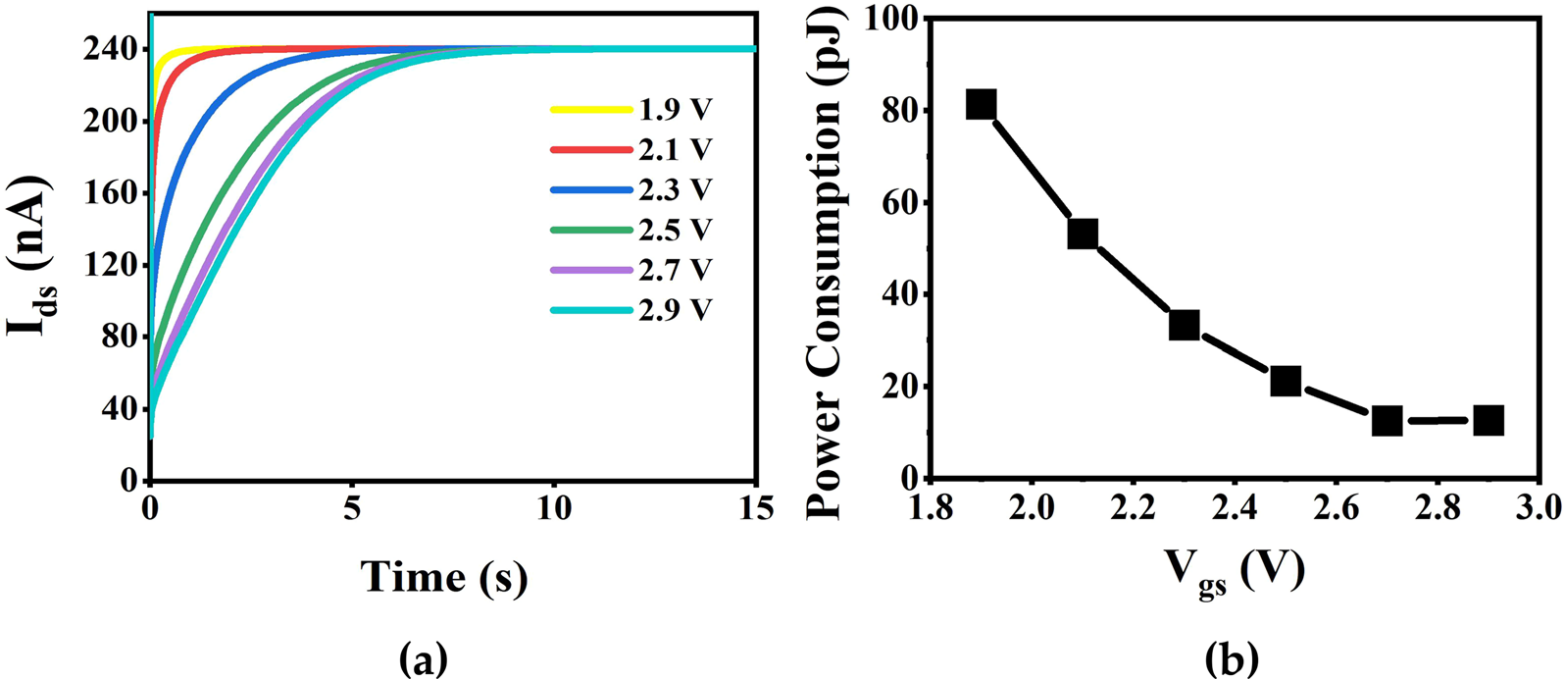
**a** The PSC with different gate voltages (1.9 V, 2.1 V, 2.3 V, 2.5 V, 2.7 V, 2.9 V) at *V*_ds_ = 0.5 V. **b** The energy consumption with different gate voltages (1.9 V, 2.1 V, 2.3 V, 2.5 V, 2.7 V, 2.9 V)

The electron affinity and band gap of zinc blende (ZB) InAs are 4.9 eV and 0.36 eV, respectively [37]. The positions of conduction band and band gaps of In_2_O_3_ and InAs NW are available from ref [38]. By applying a low gate voltage, the barriers between from conduction band to can be lowered, which make electrons captured more easily in the InAs nanowire channel. The effect of the amplitude of the gating pulse on the simulated synaptic behavior of the device was investigated. The relationship between the applied gate voltage and the excitatory PSC is shown in Fig. 6a. The excitatory PSC ranging from 1.9 to 2.9 V is 162.6 nA, 106.3 nA, 66.5 nA, 42.2 nA, 25.0 nA and 25.3 nA. As the gate voltage enhances under a low level, the barrier between the conduction band and energy level of traps is lowered, causing the electrons easier cross the barrier to be captured by the trap layer, resulting in a smaller excitatory PSC and a longer retention time. However, when the gate voltage increases to 2.9 V, the excitatory PSC abnormally increases. We attribute this phenomenon to the saturation of gate voltage regulation to depress the PSC. As shown in Fig. 2a, when the gate voltage increases to 2.9 V, *I*_ds_ is at a relatively high level. Therefore, when a gate voltage pulse of 2.9 V is applied, the capture velocity of high-speed electrons in the InAs channel and the release velocity of bound electrons in the oxide layer tend to consistent. As a result, it shows that the minimum current increases with the increase in the gate voltage. In brief, the transition from STD to LTD can be achieved by adjusting the amplitude of gate voltage.

Ultralow energy consumption is one of the most important superiorities of a neural system. In our simulation, energy consumption during a gate voltage pulse is similar as the energy consumption required to complete a neurotransmitter exchange of a biological synapse. Current synaptic devices still consume energy that is orders of magnitude greater than do biological synapses (~ 10 fJ per synaptic event). As a result, it is necessary to optimize the energy consumption of the device. In order to obtain the minimum energy consumption, we compare the PSC with the drain voltage from 2 to 0.5 V on the premise of ensuring the neurosynaptic function of the device, finally a drain voltage of 0.5 V is determined. Considering the level of gate current (*I*_gs_) is always well below 1 pA during a gate voltage pulse, the energy consumption contributed by *I*_gs_ is negligible compared with that contributed by *I*_ds_. Therefore, the formula for evaluating the energy consumption is determined by E = AIW, where A is the drain voltage, I is the current flowing across the device, and W is the width of the pulses [39]. The energy consumption at different gate voltages is shown in Fig. 6b with a drain voltage of 0.5 V. The minimum value is 12.5 uJ when the amplitude of gate voltage pulse is 2.7 V. As shown in Table 2, we compared the energy consumption of the device with other synaptic devices, which manifests that the InAs NW FET we designed has smaller energy consumption and shows better performance.

**Table 2:** Energy consumption of various synaptic devices

Devices	Energy consumption	Synaptic type	Cycle	Ref
InAs NW FET	12.5 pJ	Depression	2 ms	This work
CMOS circuit	900 pJ	Potentiation	20 ms	[16]
Memristors	10^–9^ J	Both	300 μs	[40]
Phase change memory	50 pJ	Potentiation	20 ms	[41]

## Conclusion

In this paper, we design and simulate a GAA InAs NW FET with biological synaptic behavior. The deposited In_2_O_3_ oxide layer on InAs nanowire surface acts as a trap layer capturing the hot carriers generated in the channel. Synaptic behavior is simulated in the range of low gate voltage, mainly manifested in the synaptic plasticity of STD and LTD. As the amplitude and quantity of gate voltage pulses enhance, the transition from STD to LTD can be achieved, which shows biological synaptic cooperativity. It is investigated that the state of defects of NW surface is an important factor to the simulation of synaptic function. The device exhibits good electrical properties and less energy consumption. These works demonstrate the feasibility of GAA InAs NW FETs for emerging neuromorphic networks.

## Data Availability

All data generated or analyzed during this study are included in this published article.

## References

[1] Attwell D, Laughlin SB (2001). An energy budget for signaling in the grey matter of the brain. J Cerebr Blood Flow Metab.

[2] Drachman DA (2005). Do we have brain to spare?. Neurology.

[3] Indiveri G, Liu SC (2015). Memory and information processing in neuromorphic systems. Proc IEEE.

[4] Zhu LQ, Wan CJ, Guo LQ, Shi Y, Wan Q (2014). Artificial synapse network on inorganic proton conductor for neuromorphic systems. Nat Commun.

[5] Kim K, Chen CL, Truong Q, Shen AM, Chen Y (2013). A carbon nanotube synapse with dynamic logic and learning. Adv Mater.

[6] Shi J, Ha SD, Zhou Y, Schoofs F, Ramanathan S (2013). A correlated nickelate synaptic transistor. Nat Commun.

[7] Tuma T, Pantazi A, Le Gallo M, Sebastian A, Eleftheriou E (2016). Stochastic phase-change neurons. Nat Nanotechnol.

[8] Eryilmaz SB, Kuzum D, Jeyasingh R, Kim S, BrightSky M, Lam C, Wong HSP (2014). Brain-like associative learning using a nanoscale non-volatile phase change synaptic device array. Front Neurosci-Switz.

[9] Ambrogio S, Ciocchini N, Laudato M, Milo V, Pirovano A, Fantini P, Ielmini D (2016). Unsupervised learning by spike timing dependent plasticity in phase change memory (PCM) synapses. Front Neurosci-Switz.

[10] Prezioso M, Merrikh-Bayat F, Hoskins BD, Adam GC, Likharev KK, Strukov DB (2015). Training andoperation of an integrated neuromorphic network based on metal-oxide memristors. Nature.

[11] Kim S, Du C, Sheridan P, Ma W, Choi S, Lu WD (2015). Experimental demonstration of a second-order memristor and its ability to biorealistically implement synaptic plasticity. Nano Lett.

[12] Tan ZH, Yang R, Terabe K, Yin XB, Zhang XD, Guo X (2016). Synaptic metaplasticity realized in oxide memristive devices. Adv Mater.

[13] Del Alamo JA (2011). Nanometre-scale electronics with III-V compound semiconductors. Nature.

[14] Hong CY, Yang LB, Cheng Q, Han T, Kuo JB, Chen YJ (2016). A continuous compact model incorporating higher-order correction for junctionless nanowire transistors with arbitrary doping profiles. IEEE Trans Nanotechnol.

[15] Cheng Q, Hong CY, Kuo JB, Chen YJ (2014). A surface-field-based model for nanowire mosfets with spatial variations of doping profiles. IEEE Trans Electron Devices.

[16] Indiveri G, Chicca E, Douglas R (2006). A VLSI array of low-power spiking neurons and bistable synapses with spike-timing dependent plasticity. IEEE Trans Neural Netw.

[17] Fusi S, Annunziato M, Badoni D, Salamon A, Amit DJ (2000). Spike-driven synaptic plasticity: theory, simulation, VLSI implementation. Neural Comput.

[18] Li B, Wei W, Yan X, Zhang X, Liu P, Luo YB, Zheng JH, Lu QC, Lin QM, Ren XM (2018). Mimicking synaptic functionality with an Inas nanowire phototransistor. Nanotechnology.

[19] Lee GS, Jeong JS, Yang MK, Song JD, Lee YT, Ju H (2021). Non-volatile memory behavior of interfacial InO_x_ layer in InAs nano-wire fieldeffect transistor for neuromorphic application. Appl Surf Sci.

[20] Fahad HM, Smith CE, Rojas JP, Hussain MM (2011). Silicon nanotube field effect transistor with core-shell gate stacks for enhanced high-performance operation and area scaling benefits. Nano Lett.

[21] Guo N, Hu WD, Liao L, Yip S, Ho JC, Miao JS, Zhang Z, Zou J, Jiang T, Wu SW, Chen XS, Lu W (2014). Anomalous and highly efficient InAs nanowire phototransistors based on majority carrier transport at room temperature. Adv Mater.

[22] Hofmann DM, Pfisterer D, Sann J (2007). Properties of the oxygen vacancy in ZnO. Appl Phys A.

[23] Yang YM, Peng XY, Kim HS, Kim T, Jeon S, Kang HK, Choi W, Song JD, Doh YJ, Yu D (2015). Hot carrier trapping induced negative photoconductance in Inas nanowires toward novel nonvolatile memory. Nano Lett.

[24] Nazarzadehmoafi M, Machulik S, Neske F (2014). Schottky contact by Ag on In_2_O_3_ (111) single crystals. Appl Phys Lett.

[25] Tanaka I, Tatsumi K, Nakano M (2002). First-principles calculations of anion vacancies in oxides and nitrides. J Am Ceram Soc.

[26] Goswami T, Mondal A, Singh P (2015). In_2-X_O_3-Y_ 1D perpendicular nanostructure arrays as ultraviolet detector. Solid State Sci.

[27] Gluschke JG (2018). Achieving short high-quality gate-all-around structures for horizontal nanowire field-effect transistors. Nanotechnology.

[28] Lim JK et al (2010) Alignment strategies for the assembly of nanowires with submicron diameters. Small 6:1736–1740 10.1002/smll.201000815 20665631

[29] Yamaguchi M, Yamamoto A, Sugiura H, Uemura C (1982). Thermal oxidation of InAs and characterization of the oxide film. Thin Solid Films.

[30] Li JS, Yan X, Sun FK, Zhang X, Ren XM (2015). Anomalous photoconductive behavior of a single inas nanowire photodetector. Appl Phys Lett.

[31] Voglis G, Tavernarakis N (2006). The role of synaptic ion channels in synaptic plasticity. Embo Rep.

[32] Zhu XJ, Du C, Jeong Y, Lu WD (2017). Emulation of synaptic metaplasticity in memristors. Nanoscale.

[33] Zucker RS, Regehr WG (2002). Short-term synaptic plasticity. Annu Rev Physiol.

[34] Ambrogio S, Balatti S, Nardi F, Facchinetti S, Ielmini D (2013) Spike-timing dependent plasticity in a transistor-selected resistive switching memory. Nanotechnology 24 10.1088/0957-4484/24/38/384012 23999495

[35] Li SZ, Zeng F, Chen C, Liu HY, Tang GS, Gao S, Song C, Lin YS, Pan F, Guo D (2013). Synaptic plasticity and learning behaviours mimicked through Ag interface movement in an Ag/conducting polymer/Ta memristive system. J Mater Chem C.

[36] King PDC, Veal TD, Payne DJ, Bourlange A, Egdell RG, McConville CF (2008). Surface electron accumulation and the charge neutrality level in In_2_O_3_. Phys Rev Lett.

[37] Spicer WE, Lindau I, Skeath P (1980). Unified mechanism for Schottky-Barrier formation and III-V oxide interface states. Phys Rev Lett.

[38] Pashley MD, Haberern KW, Feenstra RM, Kirchner PD (1993) Different Fermi-Level Pinning Behavior on N- and P-Type Gaas(001). Phys Rev B 48:4612–4615 10.1103/physrevb.48.4612 10008943

[39] Xu W, Min SY, Hwang H, Lee TW (2016). Organic core-sheath nanowire artificial synapses with femtojoule energy consumption. Sci Adv.

[40] Chang T, Jo SH, Lu W (2011). Short-term memory to long-term memory transition in a nanoscale memristor. ACS Nano.

[41] Kuzum D, Jeyasingh RGD, Lee B, Wong HSP (2012). Nanoelectronic programmable synapses based on phase change materials for brain-inspired computing. Nano Lett.

